# The Global Neurodegeneration Proteomics Consortium ‐ Biomarker and Drug Target Discovery Across >40,000 Biosamples for AD, PD, ALS, FTD, and Aging

**DOI:** 10.1002/alz.095579

**Published:** 2025-01-09

**Authors:** Martin Bringmann, Farhad Imam, Varsha Krish

**Affiliations:** ^1^ Johnson&Johnson, Spring House, PA USA; ^2^ Gates Ventures, Seattle, WA USA

## Abstract

**Background:**

Limited understanding of biological mechanisms behind the onset and progression of Neurodegenerative Disorders has been a burden for the discovery of novel biomarkers and treatments. Large, harmonized, patient‐derived datasets will be key in unraveling the complex biology leading to neurodegeneration. The Global Neurodegeneration Proteomics Consortium (GNPC) is a major biomarker discovery effort to unite and expand the available proteomic data for thousands of patient samples from leading dementia cohorts from around the world and comprises, to our knowledge, the largest discovery proteomics dataset to date.

**Method:**

The GNPC has brought together over 40,000 samples from over 20 different international cohorts to create and collaborate on a fully anonymized Harmonized Dataset (HDS). The HDS spans multiple neurodegenerative disorders including Alzheimer’s disease, Parkinson’s disease, amyotrophic lateral sclerosis (ALS) and frontotemporal dementia (FTD). Secure access to GNPC data is facilitated through the Alzheimer’s Disease Data Initiative ‘s online platform, the AD Workbench. De‐identified datasets submitted by each participating cohort were harmonized and further anonymized by professional data vendors. After one year of intra‐consortium analysis, the HDS will be accessible by the broader research community as a shared, global resource.

**Result:**

The first version of the Harmonized Dataset (HDS) includes over 40,000 unique samples on the SomaScan 5k and/or 7k proteomics array platform. All samples are accompanied by >40 phenotypic indicators including demographic, cognitive data, clinical scores, and disease‐specific genotype. Central hosting of de‐identified and harmonized data within the secure cloud environment provides the necessary data security, access to scalable compute resources, and facilitated collaboration between participants in diverse geographies and regulatory environments.

**Conclusion:**

With over 300,000,000 unique protein measures, the GNPC represents the largest protein biomarker discovery effort for neurodegenerative diseases to date. Initial research underway is organized via several core workstreams, including cross‐sectional and longitudinal profiling, proteogenomic mapping, multivariate prediction, and combinatorial analysis across a single disease area.

